# Extracorporeal Shock Waves Increase Markers of Cellular Proliferation in Bronchial Epithelium and in Primary Bronchial Fibroblasts of COPD Patients

**DOI:** 10.1155/2020/1524716

**Published:** 2020-08-08

**Authors:** Antonino Di Stefano, Roberto Frairia, Fabio L. M. Ricciardolo, Isabella Gnemmi, Antonella Marino Gammazza, Alessio Piraino, Francesco Cappello, Bruno Balbi, Maria Graziella Catalano

**Affiliations:** ^1^Divisione di Pneumologia e Laboratorio di Citoimmunopatologia dell'Apparato Cardio Respiratorio, Istituti Clinici Scientifici Maugeri, IRCCS, Novara, Veruno, Italy; ^2^Dipartimento di Scienze Mediche, Università di Torino, Turin, Italy; ^3^Department of Clinical and Biological Sciences, University of Torino, Turin, Italy; ^4^Dipartimento di Biomedicina Sperimentale e Neuroscienze Cliniche, Sezione di Anatomia Umana, Università di Palermo, Palermo, Italy; ^5^Euro-Mediterranean Institute of Science and Technology (IEMEST), Palermo, Italy; ^6^Respiratory Area, Chiesi Farmaceutici, Parma, Italy

## Abstract

Chronic obstructive pulmonary disease (COPD) is due to structural changes and narrowing of small airways and parenchymal destruction (loss of the alveolar attachment as a result of pulmonary emphysema), which all lead to airflow limitation. Extracorporeal shock waves (ESW) increase cell proliferation and differentiation of connective tissue fibroblasts. To date no studies are available on ESW treatment of human bronchial fibroblasts and epithelial cells from COPD and control subjects. We obtained primary bronchial fibroblasts from bronchial biopsies of 3 patients with mild/moderate COPD and 3 control smokers with normal lung function. 16HBE cells were also studied. Cells were treated with a piezoelectric shock wave generator at low energy (0.3 mJ/mm^2^, 500 pulses). After treatment, viability was evaluated and cells were recultured and followed up for 4, 24, 48, and 72 h. Cell growth (WST-1 test) was assessed, and proliferation markers were analyzed by qRT-PCR in cell lysates and by ELISA tests in cell supernatants and cell lysates. After ESW treatment, we observed a significant increase of cell proliferation in all cell types. C-Kit (CD117) mRNA was significantly increased in 16HBE cells at 4 h. Protein levels were significantly increased for c-Kit (CD117) at 4 h in 16HBE (*p* < 0.0001) and at 24 h in COPD-fibroblasts (*p* = 0.037); for PCNA at 4 h in 16HBE (*p* = 0.046); for Thy1 (CD90) at 24 and 72 h in CS-fibroblasts (*p* = 0.031 and *p* = 0.041); for TGF*β*1 at 72 h in CS-fibroblasts (*p* = 0.038); for procollagen-1 at 4 h in COPD-fibroblasts (*p* = 0.020); and for NF-*κ*B-p65 at 4 and 24 h in 16HBE (*p* = 0.015 and *p* = 0.0002). In the peripheral lung tissue of a representative COPD patient, alveolar type II epithelial cells (TTF‐1+) coexpressing c-Kit (CD117) and PCNA were occasionally observed. These data show an increase of cell proliferation induced by a low dosage of extracorporeal shock waves in 16HBE cells and primary bronchial fibroblasts of COPD and control smoking subjects.

## 1. Background

The progressive chronic airflow limitation in chronic obstructive pulmonary disease (COPD) is due to two major pathological processes: (i) remodeling and narrowing of small airways and (ii) destruction of the lung parenchyma with loss of the alveolar attachments as a result of pulmonary emphysema [1]. Chronic inflammation in the lung plays a central role in both the small airway remodeling and the pulmonary emphysema [2–4]. Lung volume reduction surgery and lung transplantation, while possible in end-stage COPD, are restricted to just a few, selected patients [5] (http://www.goldcopd.com). Regenerative therapy for COPD includes mesenchymal stromal cell (MSC) or tissue engineering therapies. But, while bone marrow MSC or adipose tissue MSC treatments showed promising results in mice with induced emphysema [6], clinical trials performed in COPD patients have been discouraging [6, 7]. There are a large number of animal studies in which lung regeneration has been successfully stimulated. For instance, in a rat model of elastase-induced emphysema, administration of all-trans RA (ATRA) stimulated alveolar regeneration [8]; keratinocyte growth factor (KGF, FGF7) administered after pneumonectomy augmented alveolarization [9]; administration of HGF stimulated alveolar regeneration, enhanced lung vascularization, and improved exercise tolerance and gas exchange [10]; intratracheal administration of bFGF to rats and dogs with elastase-induced emphysema improved alveolar dimensions and lung microvessel density [11]; and VEGF administration enhanced postpneumonectomy alveolar growth in mice [12]. But again, the attempts to stimulate lung regeneration in COPD patients with emphysema with orally administered ATRA yielded no differences in computed tomography (CT), lung function, or quality of life scores between treatment groups [13, 14], and RAR-*γ* selective agonist administration also showed no differences in CT scores or lung function in treated vs. nontreated COPD patients [15, 16]. However, the therapeutic potential of regenerative pharmacology is still at the beginning of its development. And many authors have shown that the human lung also in adulthood retains a significant regenerative potential from the large to the small airways and in terminal and respiratory bronchioles [17] and that tissue regeneration is achieved in two ways, by proliferation of common differentiated cells and/or by deployment of specialized stem/progenitor cells [18, 19].

Extracorporeal shock wave therapy (ESWT) is applied in many musculoskeletal diseases and in regenerative medicine based on its capability to induce neoangiogenesis, osteogenesis, regeneration, and remodeling through stem cell stimulation [20]. ESW in combination with tenogenic medium improved the differentiation of human adipose-derived stem cells (hASCs) into tenoblast-like cells [21]. ESW combined with osteogenic medium increased the osteogenic differentiation of treated hASCs [22], while stem cell differentiation into myofibroblasts was partially reduced by ESW treatment [23]. But, to our knowledge, no data are available on ESW treatment of primary bronchial fibroblasts of patients with COPD and control healthy smokers or bronchial epithelial cells (16HBE).

Markers of cell proliferation include CD117 (c-Kit or SCFR), a receptor tyrosine kinase protein that binds to stem cell factor (SCF), expressed on hematopoietic stem cells. It can also be expressed by mast cells, melanocytes in the skin, interstitial cells of Cajal in the digestive and urogenital tract [24], cardiac pericytes [25], amniotic fluid stem cells [26], stem/progenitor cells in conducting airway epithelium of porcine lung [27], and dendritic cells in the lung [28]. Another marker of cell proliferation is proliferating cell nuclear antigen (PCNA). It is expressed in the nuclei of cells and is involved in DNA replication, DNA repair, and chromatin remodeling [29, 30]. In the lung of COPD patients, alveolar type II epithelial cells and endothelial cells [31] and small airway bronchiolar epithelium [32] express decreased PCNA levels compared with related non-COPD control groups. A third marker of cell proliferation is CD90 (Thy1, thymocyte differentiation antigen-1), a glycophosphatidylinositol cell surface protein expressed by thymocytes, CD34+ cells, mesenchymal stem cells, endothelial cells, and cardiac fibroblasts. It is also considered a marker of multipotent mesenchymal stem cells when expressed in association with other markers (CD29, CD44, CD73, CD105) [33, 34].

We aimed in this study to analyze the proliferative effect of shock waves when applied as an external challenge to primary bronchial fibroblasts of COPD patients and control smokers, and to immortalized bronchial epithelial cells (16HBE). To this end, we investigated cell markers expression related to this proliferative stimulus.

## 2. Methods

### 2.1. Ethics Statement

Collection and processing of bronchial biopsies at the Institute of Veruno (NO) and collection and processing of the peripheral lung tissues at the University Hospital of Orbassano during lung resection for a solitary peripheral neoplasm were approved by the ethics and technical committees of the Istituti Clinici Scientifici Maugeri (CTS: p102), and San Luigi Hospital, Orbassano (TO) (CE: N. 9544, 134/2018), Italy; the study complied with the Declaration of Helsinki, and written informed consent was obtained from each participant.

### 2.2. Cell Culture and Treatments

We used the SV40 large T antigen-transformed 16HBE cell line, which retains the differentiated morphology and function of normal human bronchial epithelial cells (NHBE) [35], and primary human bronchial fibroblasts obtained from bronchial biopsies of patients with COPD (*n* = 3) and control smoking subjects (*n* = 3) with normal lung function. Primary bronchial fibroblasts were obtained from bronchial biopsies obtained for different protocol studies [36]. Bronchial biopsies were treated with type II collagenase (5 min at 37°C) (Invitrogen-GIBCA 17101.015) and cultured in DMEM until confluent primary fibroblasts were obtained. 16HBE cells and primary bronchial fibroblasts were maintained in Dulbecco's modified minimum essential medium (DMEM), supplemented with 10% v/v fetal bovine serum (FBS), 50 IU/mL penicillin, 50 *μ*g/mL streptomycin, 1x nonessential amino acids, 1 mM sodium pyruvate, and 2 mM glutamine (37°C, 5% CO_2_) [37]. When cells were 60–70% confluent, the complete medium was replaced with DMEM with 1% FBS for starvation time (24 h). The shockwave generator utilized for the *in vitro* experiments was a piezoelectric device (Piezoson 100; Richard Wolf, Knittlingen, Germany) designed for clinical use in orthopedics and traumatology. Aliquots of 1 mL of cell suspension adjusted to 1 × 10^6^ cells/mL were placed in 20 mm polypropylene tubes, completely filled with culture medium. The shock wave unit was kept in contact with the cell-containing tube by means of a water-filled cushion. Common ultrasound gel was used as a contact medium between the cushion and tube. ESW treatment was as follows: energy flux density (EFD) = 0.3 mJ/mm^2^, 500 pulses (frequency = 4 shocks/s). This EFD is a medium-high energy, we already used for previous in vitro differentiation studies in tendons [21]. After treatment, cell viability was evaluated by trypan blue exclusion and primary fibroblasts were passaged in DMEM complete for 0, 24, 48, 72 hours. 16HBE cells were cultured for 0, 24, and 48 h because of their lower resistance to starvation. T0 corresponds to 4 hours post ESW treatment for all experiments reported. Nontreated fibroblasts or 16HBE cells were used as controls. Cell growth was evaluated by the colorimetric test WST-1. All experiments were performed in quadruplicate, i.e., four independent experiments for each type of treatment (ESW or no-ESW) and each time exposure.

### 2.3. Extraction and Quantification of RNA and qRT-PCR from Primary Bronchial Fibroblasts and 16HBE

Total RNA from treated and nontreated cells was purified and isolated using an RNAspin Mini RNA Isolation kit (GE Healthcare, Life Sciences, Pittsburgh, USA) following the manufacturer's instructions. Total RNA was resuspended in 100 *μ*L nuclease-free water. RNA concentration was determined using a UV/visible spectrophotometer (*λ*260/280 nm, Eppendorf BioPhotometer plus) and stored at −80°C.

The expression of genes of interest was measured using SYBR Green (Qiagen, UK) for qPCR in a Corbett Rotor Gene 6 (Corbett, Cambridge, UK) system. One-step real-time PCR was carried out by amplifying mRNA using the QuantiFast™ SYBR Green RT-PCR kit (Qiagen, IT) according to the manufacturer's instructions and the gene specific primers (Qiagen, IT). We detected the expression of c-Kit or SCFR (CD117) (Cat. QT01844549, Qiagen), PCNA (Cat. QT00024633), Thy1 (CD90) (Cat. QT00023569), TGF*β*1 (Cat. QT00000728), Procollagen-I (Cat. QT01005725), and NF-*κ*B-p65 (Cat. QT01007370). We performed independent experiments and quantitative PCR measurements in quadruplicate for each type of treatment (ESW or no-ESW) and each time exposure. Briefly, the PCR reaction mix, prepared in a total volume of 25 *μ*L, was run on the Rotor Gene Q (Qiagen, IT) and the following PCR run protocol was used: 55°C for 10 min (reverse transcription); 95°C for 5 min (PCR initial activation step); 40 amplification cycles of 95°C for 5 s (denaturation); and 60°C for 10 s (combined annealing/extension), followed by melting curve analysis to ensure the specificity of PCR amplification. Glyceraldehyde 3 phosphate dehydrogenase (GAPDH) (QT01192646; Qiagen) was used as the reference gene for every target gene per sample, and the data were normalized against the respective GAPDH signaling. Cycle threshold (CT) values were determined using the Rotor Gene Q software (Rotor-Gene Q Series Software 2.0.2). The expression levels of all genes studied were normalized to GAPDH levels in each sample to determine the expression between treated and nontreated cells using the 2^−ΔCt^ method [38] for primary bronchial fibroblasts and the 2^−ΔΔCt^ for 16HBE cells [38].

### 2.4. ELISA Tests in the Supernatants or Cell Lysates of ESW-Treated and Nontreated Cells

Protein extraction and quantification in the supernatants or cell lysates of ESW-treated and nontreated cells were performed as reported in Table 1. Suppliers, Cat. Numbers, dilution conditions, and detection limits of the ELISA kits used are also reported. The ELISA kits, WST-1 cell proliferation kit, and M-PER mammalian protein extraction kit were used according to the manufacturer's instructions (Table 2). C-Kit (CD117), PCNA, and NF-*κ*B-p65 were quantified in cell lysates, CD90, TGF*β*1, and procollagen-1 were quantified in the cell supernatants.

### 2.5. Immunohistochemistry of the Lung Parenchyma of Patients with COPD

Samples were frozen in liquid nitrogen precooled isopentane after embedding in OCT and used for cryostat sectioning and immunostaining of some cell-proliferation-related molecules. Single immunostainings of frozen sections were performed with mouse anti–thyroid transcription factor-1 (TTF-1) (sc-53136; Santa Cruz), rabbit anti-c-Kit (CD117) (ARG51826; ARGBIO), and rabbit anti-PCNA (PAS-27214; Thermo Fisher) primary antibodies. Antibody binding was demonstrated with secondary antibodies anti-mouse (Vector, BA 2000) and anti-rabbit (Vector, BA 1000) followed by ABC kit AP (AK-5000; VECTASTAIN) and Fast-Red Substrate (red color). Double stainings were performed using also ABC kit Elite (PK-6100, VECTASTAIN), and diaminobenzidine substrate (brown color) for identification of TTF-1 positive (alveolar type II epithelial cells) [39] coexpressing c-Kit (CD117) or PCNA antigens. Slides were included in each staining run using human tonsil, nasal polyp, or breast cancer, as positive controls. For the negative control slides, normal nonspecific mouse or rabbit immunoglobulins (Santa Cruz Biotechnology, Santa Cruz, CA, USA) were used at the same protein concentration as the primary antibodies.

### 2.6. Statistical Analysis

Group data were expressed as mean ± standard deviation or median (range) or interquartile range (IQR) for morphologic-histologic data. Differences between treatment groups were analyzed using the unpaired *t*-test. Probability values of *p* < 0.05 were considered significant. Data analysis was performed using the Stat View SE Graphics program (Abacus Concepts Inc., Berkeley, CA, USA).

## 3. Results

### 3.1. ESW Effects on Cell Proliferation

**Figure 1:**
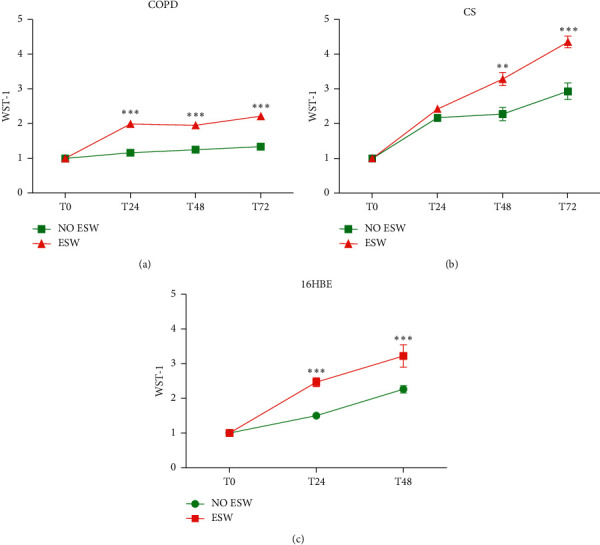
WST-1 test for evaluation of cell proliferation after extracorporeal shock wave (ESW) stimulation of primary bronchial fibroblasts of COPD patients (*n* = 3) (a), primary bronchial fibroblasts of control smokers (*n* = 3) (b), and bronchial epithelial cells (16HBE) (c). Increased cell proliferation was observed in all cellular types studied after challenge with ESW. *T*-test: ^*∗∗*^*p* < 0.01 and ^*∗∗∗*^*p* < 0.0001.

ESW treatment at a dosage of 0.3 mJ/mm^2^, 500 pulses (frequency = 4 shocks/s), of primary bronchial fibroblasts from COPD patients (*n* = 3) showed a significantly increased proliferation index at 24, 48, and 72 h after treatment compared with nontreated bronchial fibroblasts (Figure 1(a)). ESW-treated primary bronchial fibroblasts from control smokers with normal lung function (*n* = 3) also showed a significant increase of the proliferation index at 48 and 72 h after treatment (Figure 1(b)). Treated bronchial epithelial cells (16HBE) showed significantly increased proliferation index values at 24 and 48 h after treatment when compared with nontreated 16HBE cells (Figure 1(c)).

### 3.2. ESW Effects on mRNA and Protein Levels of Cell Proliferation and Cell Remodeling Markers

**Figure 2:**
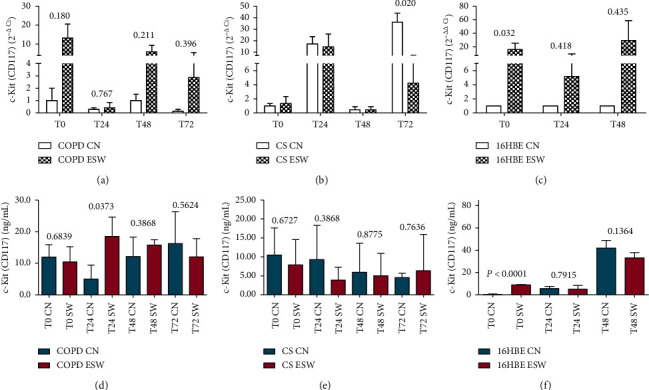
C-Kit (CD117) mRNA (a, b, c) and protein (d, e, f) expression after ESW treatment in primary bronchial fibroblasts of COPD patients (a, d), primary bronchial fibroblasts of control smokers (b, e), and bronchial epithelial cells (c, f). In bronchial epithelium (16HBE) c-Kit increased at mRNA (c) and protein (f) levels. In primary bronchial fibroblasts of COPD patients, c-Kit increased at protein level (d). *T*-test was used for comparative purposes, and *p* values are reported in the graphs.

**Figure 3:**
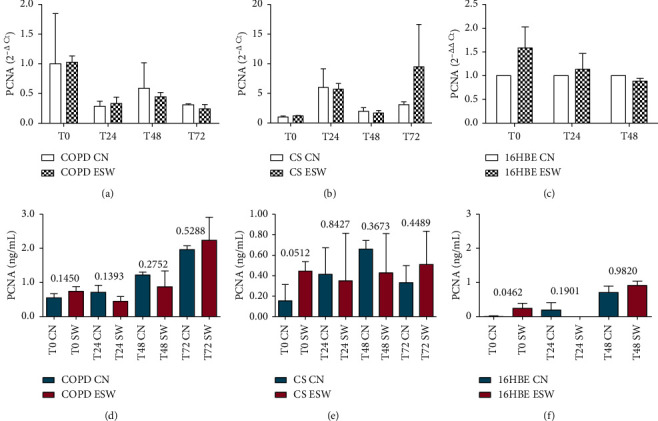
Proliferating cell nuclear antigen (PCNA) mRNA (a, b, c) and protein (d, e, f) expression after ESW treatment in primary bronchial fibroblasts of COPD patients (a, d), primary bronchial fibroblasts of control smokers (b, e), and bronchial epithelial cells (c, f). In bronchial epithelium (16HBE), PCNA increased at protein (f) level. *T*-test was used for comparative purposes, and *p* values are reported in the graphs.

**Figure 4:**
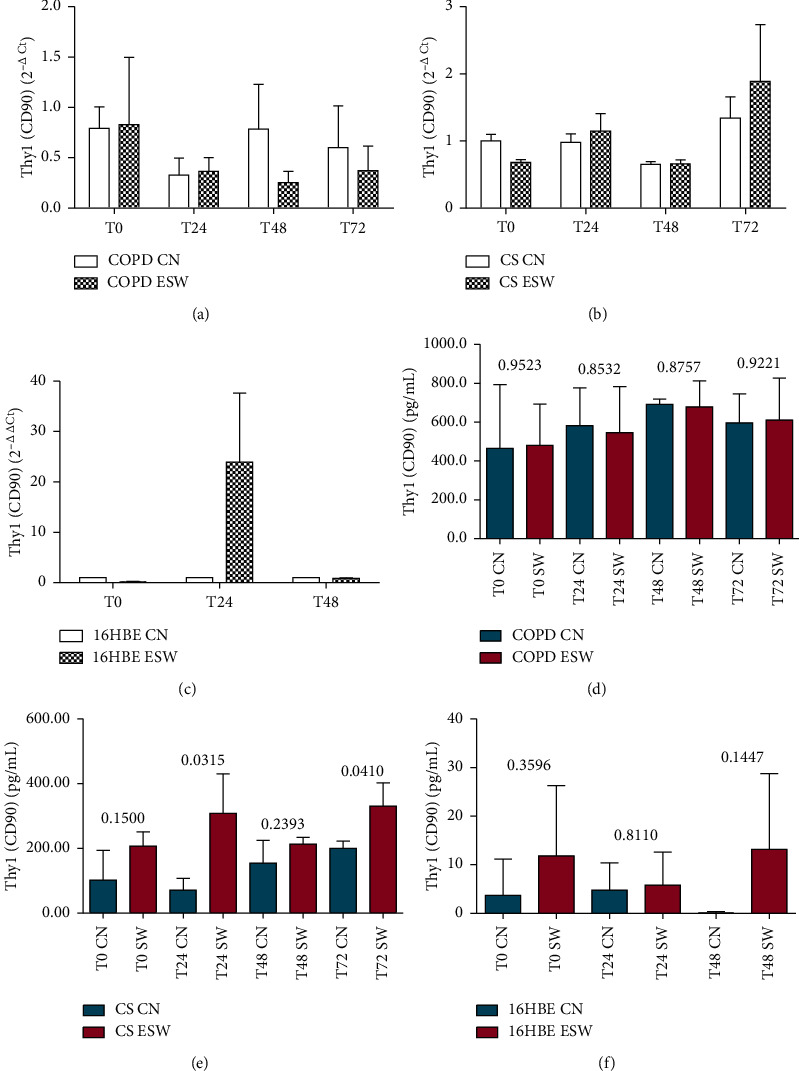
Thy1 (CD90) mRNA (a, b, c) and protein (d, e, f) expression after ESW treatment in primary bronchial fibroblasts of COPD patients (a, d), primary bronchial fibroblasts of control smokers (b, e), and bronchial epithelial cells (c, f). In primary bronchial fibroblasts of control smokers, Thy1 increased at protein level at 24 and 72 h (e). *T*-test was used for comparative purposes, and *p* values are reported in the graphs.

**Figure 5:**
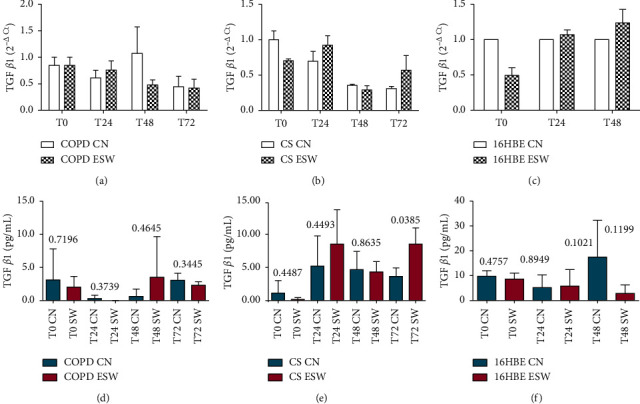
TGF*β*1 mRNA (a, b, c) and protein (d, e, f) expression after ESW treatment in primary bronchial fibroblasts of COPD patients (a, d), primary bronchial fibroblasts of control smokers (b, e), and bronchial epithelial cells (c, f). In primary bronchial fibroblasts of control smokers, TGF*β*1 increased at protein level at 72 h (e). *T*-test was used for comparative purposes, and *p* values are reported in the graphs.

**Figure 6:**
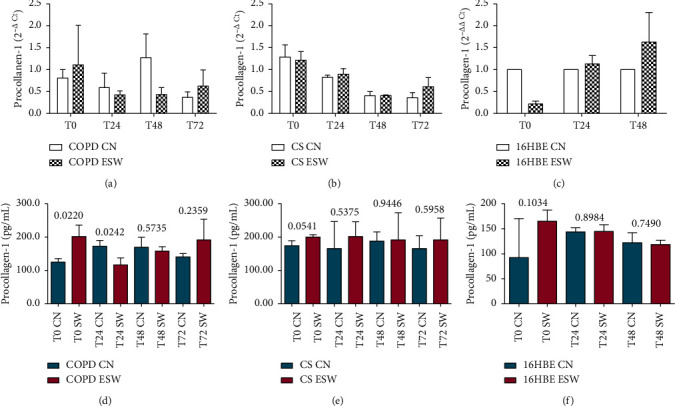
Procollagen-1 mRNA (a, b, c) and protein (d, e, f) expression after ESW treatment in primary bronchial fibroblasts of COPD patients (a, d), primary bronchial fibroblasts of control smokers (b, e), and bronchial epithelial cells (c, f). In primary bronchial fibroblasts of COPD patients, procollagen-1 increased at protein level ( d) at 4 h (T0), followed by a decrease at 24 h (panel d). *T*-test was used for comparative purposes, and *p* values are reported in the graphs.

**Figure 7:**
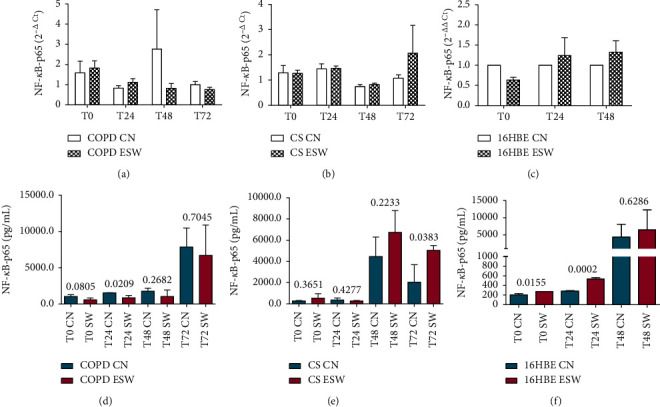
NF-*κ*B-p65 mRNA (a, b, c) and protein (d, e, f) expression after ESW treatment in primary bronchial fibroblasts of COPD patients (a, d), primary bronchial fibroblasts of control smokers (b, e), and bronchial epithelial cells (c, f). In bronchial epithelium (16HBE), NF-*κ*B-p65 increased at protein (panel f) level at 4 and 24 h of exposure. In primary bronchial fibroblasts of COPD patients, NF-*κ*B-p65 decreased at protein level (d) at 24 h. In primary bronchial fibroblasts of control smokers, NF-*κ*B-p65 increased at protein level ( e) at 72 h. *T*-test was used for comparative purposes, and *p* values are reported in the graphs.

Primary bronchial fibroblasts from COPD patients (*n* = 3), control smokers (*n* = 3), and human bronchial epithelial cells (16HBE) were stimulated with extracorporeal shock waves at a dosage of 0.3 mJ/mm^2^, 500 pulses, and compared with paired nonstimulated primary bronchial fibroblasts and 16HBE cells. C-Kit mRNA was significantly increased in ESW-treated 16HBE cells at 4 h (*p* = 0.0324) and decreased in CS-fibroblasts at 72 h (*p* = 0.020) compared with nontreated cells (Figures 2(b) and 2(c)). Furthermore, a tendency to increased c-Kit mRNA levels was observed after ESW treatment for COPD-fibroblasts (Figure 2(a)). C-Kit protein was significantly increased in the cell lysates at 24 h after ESW treatment in primary bronchial fibroblasts of COPD patients (*p* = 0.0373) (Figure 2(d)) and in 16HBE cells (*p* < 0.0001) at 4 h after ESW treatment (Figure 2(f)). No significant changes were observed for c-Kit protein in ESW-treated primary bronchial fibroblasts from control smokers (CS) with normal lung function (Figure 2(e)). PCNA mRNA levels were not significantly changed in ESW-treated fibroblasts and 16HBE cells when compared with nontreated cells (Figures 3(a)–3(c)). PCNA protein in the cell lysates showed a tendency to be increased in primary bronchial fibroblasts of CS (*p* = 0.0512) at 4 h after ESW treatment (Figure 3(e)), and a significant increase was observed at 4 h (T0) in 16HBE cells (*p* = 0.0462) after ESW treatment (Figure 3(f)). No significant changes were observed in primary bronchial fibroblasts of COPD patients (Figure 3(d)). Thy1 (CD90) mRNA levels were not significantly different in ESW-treated fibroblasts and 16HBE cells compared with nontreated cells (Figures 4(a)–4(c)). Thy1 (CD90) protein in the cell supernatants was significantly increased in primary bronchial fibroblasts of CS at 24 h (*p* = 0.0315) after ESW treatment (Figure 4(e)). No significant changes were observed in primary bronchial fibroblasts of COPD patients or in 16HBE cells (Figures 4(d) and 4(f)). TGF*β*1 mRNA levels were not significantly changed in ESW-treated fibroblasts and 16HBE cells when compared with nontreated cells (Figures 5(a)–5(c)). TGF*β*1 protein in the cell supernatants was significantly increased in primary bronchial fibroblasts of CS at 72 h (*p* = 0.0385) after ESW treatment (Figure 5(e)). No significant changes were observed in primary bronchial fibroblasts of COPD patients or in 16HBE cells (Figures 5(d) and 5(f)). Procollagen-1 mRNA levels were not significantly different in ESW-treated fibroblasts and 16HBE cells compared with nontreated cells (Figures 6(a)–6(c)). Procollagen-1 protein in the cell supernatants was significantly increased in primary bronchial fibroblasts of COPD patients at 4 h (*p* = 0.0220) after ESW treatment (Figure 6(d)). No significant changes were observed in primary bronchial fibroblasts of CS or in 16HBE cells (Figures 6(e) and 6(f)). NF-*κ*B-p65 mRNA levels were not significantly changed in ESW-treated fibroblasts and 16HBE cells when compared with nontreated cells (Figures 7(a)–7(c)). NF-*κ*B-p65 protein in the cell lysates was decreased in primary bronchial fibroblasts of COPD patients at 24 h (*p* = 0.0209) after ESW treatment (Figure 7(d)) and increased in 16HBE cells at 4 h (*p* = 0.0155) and 24 h (*p* = 0.0002) after ESW treatment (Figure 7(f)). No significant changes were observed in primary bronchial fibroblasts of CS (Figure 7(e)).

### 3.3. Immunohistochemistry in the Lung Parenchyma of COPD Patients of Alveolar Type II Epithelial Cells Expressing c-Kit and PCNA

**Figure 8:**
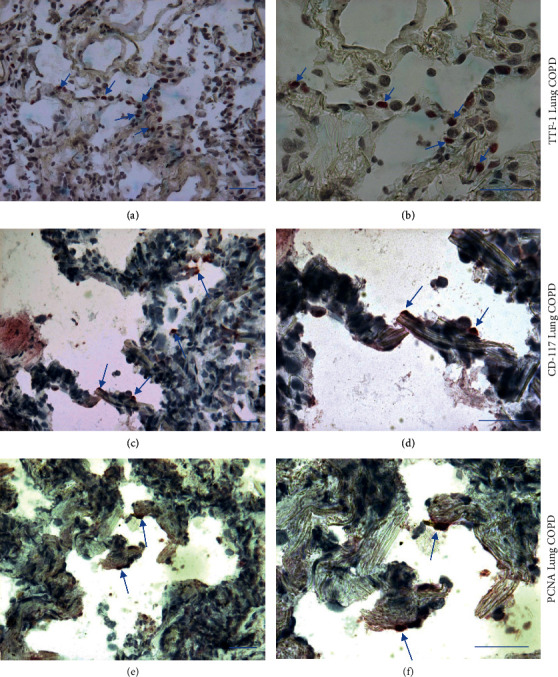
Photomicrographs showing thyroid transcription factor-1 (TTF-1) expression (panels a, b), c-Kit (CD117) (c, d), and proliferating cell nuclear antigen (PCNA) (e, f) in the peripheral lung tissue of a representative patient with chronic obstructive pulmonary disease (COPD). Arrows indicate positively stained cells mainly located in the alveolar septa. Bars = 50 microns.

**Figure 9:**
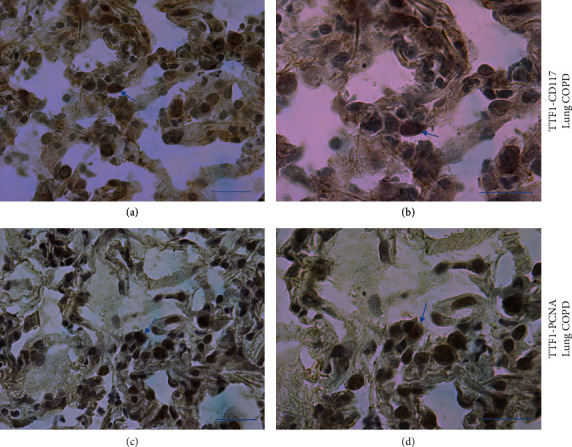
Photomicrographs showing alveolar type II epithelial cells (TTF-1+ cells, red color) coexpressing c-Kit (CD117) (brown color) (a, b) and PCNA (brown color) (c, d) in the peripheral lung tissue of a representative patient with COPD. Positive double-stained cells can be recognized in the alveolar septa, even though their presence was only rarely observed. Arrows indicate positively stained cells located in the alveolar septa. Bars = 50 microns.

In the lung parenchyma of COPD patients, alveolar type II epithelial cells were identified by the use of anti–thyroid transcription factor-1 (TTF-1) antibody. Immunopositivity for c-Kit (CD117) and PCNA was also occasionally observed in alveolar septa (Figure 8). Double staining, used for identification of TTF-1+ cells coexpressing c-Kit (CD117) (Figures 9(a) and 9(b)) and PCNA (Figures 9(c) and 9(d)), showed that alveolar type II epithelial cells coexpressing c-Kit and PCNA were present even though rarely observed.

## 4. Discussion

This study shows that extracorporeal shock waves induce cell proliferation of bronchial epithelial cells (16HBE) and primary bronchial fibroblasts of COPD patients and control smokers. As far as markers of cell proliferation are concerned, c-Kit (CD117) was increased in bronchial epithelium at both mRNA and protein levels 4 h after ESW treatment and it was also increased in primary bronchial fibroblasts at 24 h after ESW challenge. Other markers indicative of cell proliferation were also increased: PCNA protein increased in bronchial epithelial cells at 4 h after ESW challenge; Thy1 (CD90) protein increased in CS–primary bronchial fibroblasts at 24 and 72 h after ESW treatment; molecules more related to remodeling, such as TGF*β*1 protein, were increased in CS–primary bronchial fibroblasts at 72 h after ESW treatment and procollagen-1 protein increased at 4 h, followed by a decrease at 24 h, in COPD–primary bronchial fibroblasts after ESW treatment. A marker of inflammation, transcription factor NF-*κ*B-p65 protein, was decreased in COPD–primary bronchial fibroblasts at 24 h after ESW treatment, but it was increased in CS–primary bronchial fibroblasts and in bronchial epithelial cells after ESW treatment. Markers of cell proliferation such as c-Kit and PCNA were observed in the peripheral lung of COPD patients, and both these markers were occasionally coexpressed by alveolar epithelial type II cells (TTF-1+) in these patients.

Extracorporeal shock wave therapy is applied in regenerative medicine since it is capable of inducing neoangiogenesis, osteogenesis, and remodeling through stem cell stimulation [20]. On the other hand, while regenerative therapy applied to mice with induced emphysema has shown promising results [6], clinical trials performed in COPD patients were discouraging [6, 7]. Since the human lung also in adulthood maintains a significant regenerative potential [17–19], due to proliferation of differentiated of stem/progenitor cells and/or by their stimulation [18, 19], we here investigated the proliferative action of ESW at low dosage in bronchial epithelial cells and in primary bronchial fibroblasts of control smokers (CS) and patients with COPD. Our data show that all the cell types studied significantly increased their proliferation index (WST-1 test) after ESW treatment, in agreement with data previously obtained for muscle cells or tendon fibroblasts [20]. Interestingly, the c-Kit (CD117) receptor tyrosine kinase protein and mRNA were increased in 16HBE cells, and c-Kit protein also increased in primary bronchial fibroblasts of COPD patients, after ESW stimulation. It is not clear, however, if this cell response represents an intermediate dedifferentiation step or a simple pro-proliferative stimulus for stimulated 16HBE cells and COPD–primary bronchial fibroblasts. Since we exposed well-differentiated cells, we believe that this transitory increment may be interpreted as a pro-proliferative stimulus induced by ESW exposure.

In bronchial epithelial cells (16HBE), proliferating cell nuclear antigen (PCNA), considered a marker of cell proliferation, was increased after ESW stimulation, confirming again the pro-proliferative role of ESW exposure for these lung structure cells. This finding, in view of the decreased PCNA levels reported in the lung of COPD patients [31, 32] compared with control subjects, is particularly relevant, since ESW stimulation may contrast these lower PCNA levels characterizing the damaged lung of these patients.

The increased Thy-1 (CD90) protein level shown after ESW exposure in CS–primary bronchial fibroblasts was not observed in ESW-treated COPD–primary bronchial fibroblasts, or in 16HBE treated cells. PCNA protein also tended to be higher in CS–primary bronchial fibroblasts after ESW treatment but not in COPD–primary bronchial fibroblasts. These differences in the response to ESW challenge of COPD– and CS–primary bronchial fibroblasts may in part be due to the reduced proliferation capacity of these cells derived from COPD lungs, as previously reported [40, 41]. In our well-differentiated ESW-exposed fibroblasts, we interpret the increment of Thy-1 protein after ESW treatment—like that of c-Kit—as a pro-proliferative stimulus induced by the treatment.

We found increased levels of secreted TGF*β*1 in CS–primary bronchial fibroblasts 72 h after ESW stimulation. TGF*β* signaling pathways are involved in the regulation of many cell functions and in the maintenance of cellular homeostasis [42]. We recently reported a decrease of TGF*β*1 and TGF*β*3 in bronchiolar epithelium and alveolar macrophages of COPD patients compared with CS [36], and this decrease may favor the increase of autoimmunity responses in these patients [36]. We speculate that the induction through ESW challenge of an increase of TGF*β* in bronchial fibroblasts may play a role in the TGF*β* repositioning and gain in homeostatic function of this important protein in the lungs of COPD patients.

TGF*β* induced extracellular matrix and procollagen-1 production has been reported in pulmonary fibroblasts [43], even though it was also reported that the increase of profibrotic markers, including procollagen-1 in human lung fibroblasts, may be NLRP3 inflammasome dependent and TGF*β* independent [44] and associated with increased inflammation of the lung [44]. We here observed a transitory increase of procollagen-1 protein in COPD–primary bronchial fibroblasts at 4 h after ESW treatment, followed by a significant decrease at 24 h. However, we cannot exclude a modest profibrosing activity of ESW treatments when applied to bronchial fibroblasts. Interestingly, after ESW treatment, we here observed a decrement of the NF-*κ*B-p65 proinflammatory transcription factor in COPD–primary bronchial fibroblasts and an increment of this protein in CS–primary bronchial fibroblasts and in 16HBE treated cells. These conflicting results may be related to “basic” differences between CS–primary bronchial fibroblasts and COPD–primary bronchial fibroblasts showing a different response to the ESW stimulation. Speculatively, we can hypothesize that increased senescence of COPD–primary bronchial fibroblasts may influence this different response to ESW treatment [40, 41]. Quantitation of c-Kit+ (CD117) cells in the lung of COPD patients and control smokers and nonsmokers showed an occasional presence of these cells in the lung with no significant differences between COPD and control subjects [45]. PCNA levels were reported as decreased in the lungs of COPD patients compared with non-COPD control groups [32]. In agreement with these studies, we identified the presence of alveolar type II epithelial cells (TTF-1+ cells), c-Kit+, and PCNA + cells in the lung of patients with COPD. The presence of alveolar type II epithelial cells coexpressing c-Kit or PCNA was only rarely observed. A formal quantitation of these single- and double-stained cells was not performed since this is outside the scope of the present study. However, the presence of c-Kit+ and PCNA + cells in the lung and alveolar septa of COPD patients supports the notion that these cells could participate in the regenerative process induced by external stimulation (ESW) of these cells. This could be the object of future “*in vivo*” investigations using ESW stimulations of the lung, in order to verify the “*in vivo*” effects on induction of lung cell damage and proliferation. However, in this respect, as ultrasound-treated lungs of differently sized animal models showed lung hemorrhage induction at a high acoustic ultrasound exposure [46], lower acoustic ultrasound exposures or shock waves generated by piezoelectric devices need to be studied. Furthermore, differences have been reported between focused and radial ESW [47]. Focused ESW, as we used in the present study, differ in the penetration depth, physical characteristics, and generating technique [47]. Different cell types seem to be differentially influenced by radial and focused ESW [47]. In our knowledge, data on lung fibroblasts or epithelial cells challenged with radial ESW are not disposable. A second option could be the intratracheal administration of adipose-derived stem cells pretreated with ESW [22, 23, 48] in the attempt to improve the alveolar septa reparative response in mice with experimentally induced emphysema.

## 5. Conclusion

To our knowledge, this is the first study to apply extracorporeal shock waves to bronchial epithelial cells and primary bronchial fibroblasts of COPD patients in an attempt to induce cell proliferation. ESW treatment induced increased cell proliferation and an increase of specific markers of cell proliferation. Our “in vitro” study provides support for the application of ESW treatment “in vivo” in a mouse model of injured lungs with induced emphysema.

## Figures and Tables

**Table 1:** Clinical characteristics of chronic obstructive pulmonary disease (COPD) patients and control smokers who provided bronchial fibroblasts for “in vitro” experiments.

Subjects	Age (years)	M/F	Pack/years	Ex-smoker/current smoker	FEV1% pre-BD	FEV1% post-BD	FEV1/FVC%
COPD1	55	M	40	Current	56	64	46
COPD2	56	M	109	Current	40	39	36
COPD3	78	M	10	Ex	78	85	60
Mean ± SD	63 ± 13	—	53 ± 51	—	58 ± 19	63 ± 23	47 ± 12
CS1	73	M	60	Current	86	ND	74
CS2	63	M	52	Current	97	ND	77
CS3	56	M	10	Current	112	ND	86
Mean ± SD	64 ± 8	—	41 ± 27	—	98 ± 13	—	79 ± 6

Individual and mean ± standard deviation (SD) data. M: male; F: female; FEV1: forced expiratory volume in 1 s; BD: bronchodilator; FVC: forced vital capacity; ND: not determined. Patients were classified according to the Global Initiative for Chronic Obstructive Lung disease (http://goldcopd.org) levels of severity for COPD. For COPD patients, FEV1/FVC% are postbronchodilator values. ANOVA test: FEV1%, *p* = 0.039; FEV1/FVC%, *p* = 0.015. No significant differences were observed for age (*p* = 0.916) and pack/years (*p* = 0.728) smoked.

**Table 2:** List of ELISA tests, cell proliferation, and protein extraction kits used. For ELISA tests, dilution of the supernatants or cell lysate samples used and detection limits are also reported.

Target	Supplier	Cat. #^a^	Dilution	Detection limit (range)
c-Kit or SCFR (CD117)	Cloud-Clone Corp.	SEA121 Hu	1 : 5 (PBS)	0.61 ng/mL (1.56–100 ng/mL)
PCNA	Cloud-Clone Corp.	SEA591Mi	1 : 5 (PBS)	0.055 ng/mL (0.156–10 ng/mL)
Thy1 (CD90)	Cloud-Clone Corp.	SEB404 Hu	No dil	5.3 pg/mL (12.5–800 pg/mL)
TGF*β*1	Cloud-Clone Corp.	SEA124 Hu	No dil.	5.8 pg/mL (15.6–1000 pg/mL)
Procollagen-1	Cloud-Clone Corp.	SEA957 Hu	No dil.	12.3 pg/mL (31.2–2000 pg/mL)
NF-*κ*B-p65	Invitrogen	KHO0371	1 : 5 (diluent buffer)	<17pg/mL (0–5000 pg/mL)
WST-1 cell proliferation	Abnova	KA1384	//	//
M-PER mammalian protein extraction	Thermo Scientific	78501	//	//

## Data Availability

The experimental data for “in vitro” study and lung functional data of patients are available from the corresponding author upon request.

## Abbreviations

MSC: Mesenchymal stromal cell
KGF: Keratinocyte growth factor
FGF: Fibroblast growth factor
HGF: Hepatocyte growth factor
VEGF: Vascular endothelial growth factor
CT: Computed tomography
ATRA: All-trans retinoic acid
RAR: Retinoic acid receptor
ESW: Extracorporeal shock waves
ASC: Adipose-derived stem cells
SCF: Stem cell factor
PCNA: Proliferating cell nuclear antigen
Thy1: Thymocyte differentiation antigen-1
TTF-1: Thyroid transcription factor-1
TGF: Transforming growth factor.

## References

[1] Hogg J. C., Chu F., Utokaparch S. (2004). The nature of small-airway obstruction in chronic obstructive pulmonary disease. *New England Journal of Medicine*.

[2] Hogg J. C. (2004). Pathophysiology of airflow limitation in chronic obstructive pulmonary disease. *The Lancet*.

[3] Barnes P. J. (2014). Cellular and molecular mechanisms of chronic obstructive pulmonary disease. *Clinics in Chest Medicine*.

[4] Di Stefano A., Caramori G., Ricciardolo F. L., Capelli A., Adcock I. M., Donner C. F. (2004). Cellular and molecular mechanisms in chronic obstructive pulmonary disease: an overview. *Clinical and Experimental Allergy*.

[5] Slama A., Taube C., Kamler M., Aigner C. (2018). Lung volume reduction followed by lung transplantation-considerations on selection criteria and outcome. *Journal of Thoracic Disease*.

[6] Oh D. K., Kim Y. S., Oh Y. M. (2017). Lung regeneration therapy for chronic obstructive pulmonary disease. *Tuberculosis and Respiratory Diseases*.

[7] Broekman W., Khedoe P. P. S. J., Schepers K., Roelofs H., Stolk J., Hiemstra P. S. (2018). Mesenchymal stromal cells: a novel therapy for the treatment of chronic obstructive pulmonary disease?. *Thorax*.

[8] Massaro D., Massaro G. D. (2010). Lung development, lung function, and retinoids. *New England Journal of Medicine*.

[9] Kaza A. K., Kron I. L., Leuwerke S. M., Tribble C. G., Laubach V. E. (2002). Keratinocyte growth factor enhances post-pneumonectomy lung growth by alveolar proliferation. *Circulation*.

[10] Shigemura N., Sawa Y., Mizuno S. (2005). Amelioration of pulmonary emphysema by in vivo gene transfection with hepatocyte growth factor in rats. *Circulation*.

[11] Morino S., Toba T., Tao H. (2007). Fibroblast growth factor-2 promotes recovery of pulmonary function in a canine models of elastase-induced emphysema. *Experimental Lung Research*.

[12] Sakurai M. K., Lee S., Arsenault D. A. (2007). Vascular endothelial growth factor accelerates compensatory lung growth after unilateral pneumonectomy. *American Journal of Physiology-Lung Cellular and Molecular Physiology*.

[13] Mao J. T., Goldin J. G., Dermand J. (2002). A pilot study of all-trans-retinoic acid for the treatment of human emphysema. *American Journal of Respiratory and Critical Care Medicine*.

[14] Muindi J. R., Roth M. D., Wise R. A. (2008). Pharmacokinetics and metabolism of all-trans- and 13-cis-retinoic acid in pulmonary emphysema patients. *The Journal of Clinical Pharmacology*.

[15] Stolk J., Stockley R. A., Stoel B. C. (2012). Randomised controlled trial for emphysema with a selective agonist of the *γ*-type retinoic acid receptor. *European Respiratory Journal*.

[16] Ng-Blichfeldt J. P., Gosens R., Dean C., Griffiths M., Hind M. (2019). Regenerative pharmacology for COPD: breathing new life into old lungs. *Thorax*.

[17] Kotton D. N., Morrisey E. E. (2014). Lung regeneration: mechanisms, applications and emerging stem cell populations. *Nature Medicine*.

[18] Vaughan A. E., Brumwell A. N., Xi Y. (2015). Lineage-negative progenitors mobilize to regenerate lung epithelium after major injury. *Nature*.

[19] Hogan B. L., Barkauskas C. E., Chapman H. A. (2014). Repair and regeneration of the respiratory system: complexity, plasticity, and mechanisms of lung stem cell function. *Cell Stem Cell*.

[20] D’Agostino M. C., Frairia R., Romeo P. (2016). Extracorporeal shockwaves as regenerative therapy in orthopedic traumatology: a narrative review from basic research to clinical practice. *Journal of Biological Regulators & Homeostatic Agents*.

[21] Rinella L., Marano F., Paletto L. (2018). Extracorporeal shock waves trigger tenogenic differentiation of human adipose-derived stem cells. *Connective Tissue Research*.

[22] Catalano M. G., Marano F., Rinella L. (2017). Extracorporeal shockwaves (ESWs) enhance the osteogenic medium-induced differentiation of adipose-derived stem cells into osteoblast-like cells. *Journal of Tissue Engineering and Regenerative Medicine*.

[23] Rinella L., Marano F., Berta L. (2016). Extracorporeal shock waves modulate myofibroblast differentiation of adipose-derived stem cells. *Wound Repair and Regeneration*.

[24] Hashitani H., Lang R. J., Suzuki H. (2010). Role of perinuclear mitochondria in the spatiotemporal dynamics of spontaneous Ca2+ waves in interstitial cells of Cajal-like cells of the rabbit urethra. *British Journal of Pharmacology*.

[25] Iancu C. B., Rusu M. C., Mogoantă L., Hostiuc S., Grigoriu M. (2018). Myocardial telocyte-like cells: a review including new evidence. *Cells Tissues Organs*.

[26] Loukogeorgakis S. P., De Coppi P. (2017). Concise review: amniotic fluid stem cells: the known, the unknown, and potential regenerative medicine applications. *Stem Cells*.

[27] Jia Y., You X., Ma N. (2019). Phenotypic analysis of BrdU label-retaining cells during the maturation of conducting airway epithelium in a porcine lung. *Stem Cells International*.

[28] Oriss T. B., Krishnamoorthy N., Ray P., Ray A. (2014). Dendritic cell c-kit signaling and adaptive immunity: implications for the upper airways. *Current Opinion in Allergy and Clinical Immunology*.

[29] Lehmann A. R. (2006). Translesion synthesis in mammalian cells. *Experimental Cell Research*.

[30] Lehmann A. R. (2011). Ubiquitin-family modifications in the replication of DNA damage. *FEBS Letters*.

[31] Tsuji T., Aoshiba K., Nagai A. (2006). Alveolar cell senescence in patients with pulmonary emphysema. *American Journal of Respiratory and Critical Care Medicine*.

[32] Chiappara G., Gjomarkaj M., Sciarrino S., Vitulo P., Pipitone L., Pace E. (2014). Altered expression of p21, activated caspase-3, and PCNA in bronchiolar epithelium of smokers with and without chronic obstructive pulmonary disease. *Experimental Lung Research*.

[33] Ullah I., Subbarao R. B., Rho G. J. (2015). Human mesenchymal stem cells - current trends and future prospective. *Bioscience Reports*.

[34] Skurikhin E. G., Pakhomova A. V., Krupin V. A. (2016). Response of inflammatory mediators, extracellular matrix proteins and stem and progenitor cells to emphysema. *Bulletin of Experimental Biology and Medicine*.

[35] Cozens A. L., Yezzi M. J., Kunzelmann K. (1994). CFTR expression and chloride secretion in polarized immortal human bronchial epithelial cells. *American Journal of Respiratory Cell and Molecular Biology*.

[36] Di Stefano A., Sangiorgi C., Gnemmi I. (2018). TGF-*β* signaling pathways in different compartments of the lower airways of patients with stable COPD. *Chest*.

[37] Marcelino M. Y., Fuoco N. L., de Faria C. A., Kozma Rde L., Marques L. F., Ribeiro-Paes J. T. (2014). Animal models in chronic obstructive pulmonary disease-an overview. *Experimental Lung Research*.

[38] Livak K. J., Schmittgen T. D. (2001). Analysis of relative gene expression data using real-time quantitative PCR and the 2(-Delta Delta C(T)) Method. *Methods*.

[39] Vlachaki E. M., Koutsopoulos A. V., Tzanakis N. (2010). Altered surfactant protein-A expression in type II pneumocytes in COPD. *Chest*.

[40] Hamsanathan S., Alder J. K., Sellares J., Rojas M., Gurkar A. U., Mora A. L. (2019). Cellular senescence: the trojan horse in chronic lung diseases. *American Journal of Respiratory Cell and Molecular Biology*.

[41] Bartling B., Hofmann H. S. (2018). Reduced proliferation capacity of lung cells in chronic obstructive pulmonary disease. *Zeitschrift für Gerontologie und Geriatrie*.

[42] Aschner Y., Downey G. P. (2016). Transforming Growth Factor-*β*: Master Regulator of the Respiratory System in Health and Disease. *American Journal of Respiratory Cell and Molecular Biology*.

[43] Brandsma C. A., Timens W., Jonker M. R., Rutgers B., Noordhoek J. A., Postma D. S. (2013). Differential effects of fluticasone on extracellular matrix production by airway and parenchymal fibroblasts in severe COPD. *American Journal of Physiology-Lung Cellular and Molecular Physiology*.

[44] Hussain S., Sangtian S., Anderson S. M. (2014). Inflammasome activation in airway epithelial cells after multi-walled carbon nanotube exposure mediates a profibrotic response in lung fibroblasts. *Particle and Fibre Toxicology*.

[45] López-Giraldo A., Cruz T., Molins L. (2018). Characterization, localization and comparison of c-Kit+ lung cells in never smokers and smokers with and without COPD. *BMC Pulmonary Medicine*.

[46] O’Brien W. D., Yang Y., Simpson D. G. (2006). Threshold estimation of ultrasound-induced lung hemorrhage in adult rabbits and comparison of thresholds in mice, rats, rabbits and pigs. *Ultrasound in Medicine & Biology*.

[47] Hochstrasser T., Frank H. G., Schmitz C. (2016). Dose-dependent and cell type-specific cell death and proliferation following in vitro exposure to radial extracorporeal shock waves. *Scientific Reports*.

[48] Vallese D., Ricciardolo F. L., Gnemmi I. (2015). Phospho-p38 MAPK expression in COPD patients and asthmatics and in challenged bronchial epithelium. *Respiration*.

